# Understanding Lactobacillus paracasei and Streptococcus oralis Biofilm Interactions through Agent-Based Modeling

**DOI:** 10.1128/mSphere.00875-21

**Published:** 2021-12-15

**Authors:** Linda Archambault, Sherli Koshy-Chenthittayil, Angela Thompson, Anna Dongari-Bagtzoglou, Reinhard Laubenbacher, Pedro Mendes

**Affiliations:** a Center for Quantitative Medicine, University of Connecticut grid.208078.5grid.63054.34 School of Medicine, Farmington, Connecticut, USA; b Department of Oral Health and Diagnostic Sciences, University of Connecticut grid.208078.5grid.63054.34 School of Dental Medicine, Farmington, Connecticut, USA; c Department of Medicine, University of Florida, Gainesville, Florida, USA; d Center for Cell Analysis and Modeling, University of Connecticut grid.208078.5grid.63054.34 School of Medicine, Farmington, Connecticut, USA; e Department of Cell Biology, University of Connecticut grid.208078.5grid.63054.34 School of Medicine, Farmington, Connecticut, USA; University of Iowa

**Keywords:** *Lactobacillus paracasei*, *Streptococcus oralis*, agent-based modeling, biofilm, computational modeling, oral health

## Abstract

As common commensals residing on mucosal tissues, *Lactobacillus* species are known to promote health, while some Streptococcus species act to enhance the pathogenicity of other organisms in those environments. In this study, we used a combination of *in vitro* imaging of live biofilms and computational modeling to explore biofilm interactions between Streptococcus oralis, an accessory pathogen in oral candidiasis, and Lactobacillus paracasei, an organism with known probiotic properties. A computational agent-based model was created where the two species interact only by competing for space, oxygen and glucose. Quantification of bacterial growth in live biofilms indicated that S. oralis biomass and cell numbers were much lower than predicted by the model. Two subsequent models were then created to examine more complex interactions between these species, one where *L. paracasei* secretes a surfactant, and another where *L. paracasei* secretes an inhibitor of S. oralis growth. We observed that the growth of S. oralis could be affected by both mechanisms. Further biofilm experiments support the hypothesis that *L. paracasei* may secrete an inhibitor of S. oralis growth, although they do not exclude that a surfactant could also be involved. This contribution shows how agent-based modeling and experiments can be used in synergy to address multiple species biofilm interactions, with important roles in mucosal health and disease.

**IMPORTANCE** We previously discovered a role of the oral commensal Streptococcus oralis as an accessory pathogen. S. oralis increases the virulence of Candida albicans infections in murine oral candidiasis and epithelial cell models through mechanisms which promote the formation of tissue-damaging biofilms. *Lactobacillus* species have known inhibitory effects on biofilm formation of many microbes, including Streptococcus species. Agent-based modeling has great advantages as a means of exploring multifaceted relationships between organisms in complex environments such as biofilms. Here, we used an iterative collaborative process between experimentation and modeling to reveal aspects of the mostly unexplored relationship between S. oralis and *L. paracasei* in biofilm growth. The inhibitory nature of *L. paracasei* on S. oralis in biofilms may be exploited as a means of preventing or alleviating mucosal fungal infections.

## INTRODUCTION

*Lactobacillus* and Streptococcus species are ubiquitous commensals found in the human oral cavity but also the genitourinary and gastrointestinal tracts. Mitis group streptococci (MGS), primarily represented by Streptococcus oralis, Streptococcus sanguinis, Streptococcus gordonii, and Streptococcus mitis, are prominent among first colonizers of biofilms on mucosal and tooth surfaces (1, 2). MGS were originally found to play a positive role, maintaining microbiome homeostasis in the oral cavity by antagonizing other microbes such as the cariogenic Streptococcus mutans (3, 4). Although they are members of the healthy oral microbiota, MGS were more recently recognized for their role as accessory pathogens, enhancing the virulence of potentially harmful members of the microbiota, such as Candida albicans (5–8) and Porphyromonas gingivalis (9, 10). In addition, MGS can be pathogens in their own right: when they enter the bloodstream, they can cause endocarditis, bacteremia, and toxic shock (11–13).

Many *Lactobacillus* species have probiotic properties that promote gut, vaginal, and oral health (14–17). Lactobacilli possess a diverse array of mechanisms implicated in the inhibition of vital processes in other bacteria; these include inhibition of growth through production of lactic acid and bacteriocins, and prevention of attachment to surfaces by competition, coaggregation, and production of biosurfactants, which may also promote biofilm dispersion (18–22). In the oral environment, *Lactobacillus* species inhibit the growth and biofilm formation of S. mutans via multiple mechanisms. For example, secreted molecules found in supernatants of *Lactobacillus* cultures inhibit growth, adhesion, and biofilm formation (23, 24), and cell wall component lipoteichoic acid interferes with S. mutans sucrose metabolism, reducing the production of exopolysaccharide, an important component of biofilms (25). *Lactobacillus* spp. also inhibit Streptococcus pyogenes hemolytic activity and adhesion to epithelial cells (26). In addition to the great variety of antimicrobial effects attributed to different *Lactobacillus* spp., a considerable genetic and phenotypic diversity exists in oral streptococcal species, and even strains within the same species, which affects growth in different oral ecological niches and their role as pathobionts (6). Lactobacillus paracasei is known to produce molecules with antimicrobial and surfactant properties (27–29), but interactions between *L. paracasei* and S. oralis in the biofilm growth form have never been explored.

To fully understand the complex community interactions between species, mathematical modeling is a complement to an experimental approach (30, 31). It helps consolidate data, and after validation, it can help in making predictions (32) and provide an integrative and quantitative understanding of the system studied. Agent-based models (ABMs) are particularly suited to represent biofilms as they capture the activities of each individual cell (autonomous agent) in the community (33). These models incorporate rules of growth, division, movement, and decay for each cell, and these rules can be deterministic or stochastic. The cells are embedded in a spatial environment with relevant physical constraints, such as diffusion of chemicals (34, 35). The behavior of each individual agent and the environmental constraints contribute to the emergence of the total population behavior, i.e., the biofilm development and structure. Very few agent-based models have been constructed using input both from the literature and from experiments (36, 37). Our model is another addition to this small group of ABMs built through cross talk between experimentation and simulation.

This work aims to further our understanding of the interactions between S. oralis and *L. paracasei* during biofilm growth with a rarely used combination of agent-based modeling and experimentation. The agent-based model was a device to better understand the dual-species biofilm growth characteristics. The growth parameters of the model were estimated using the experimentally determined behavior of single-species biofilms and data from the literature. Live fluorescence imaging showed that the growth of S. oralis is inhibited in the dual biofilm with *L. paracasei*. We then constructed two models expressing two distinct hypotheses: noncompetitive inhibition and surfactant production. The models were validated with further experiments to explore the nature of the interactions between S. oralis and *L. paracasei*.

## RESULTS

### Model calibration.

**FIG 1:**
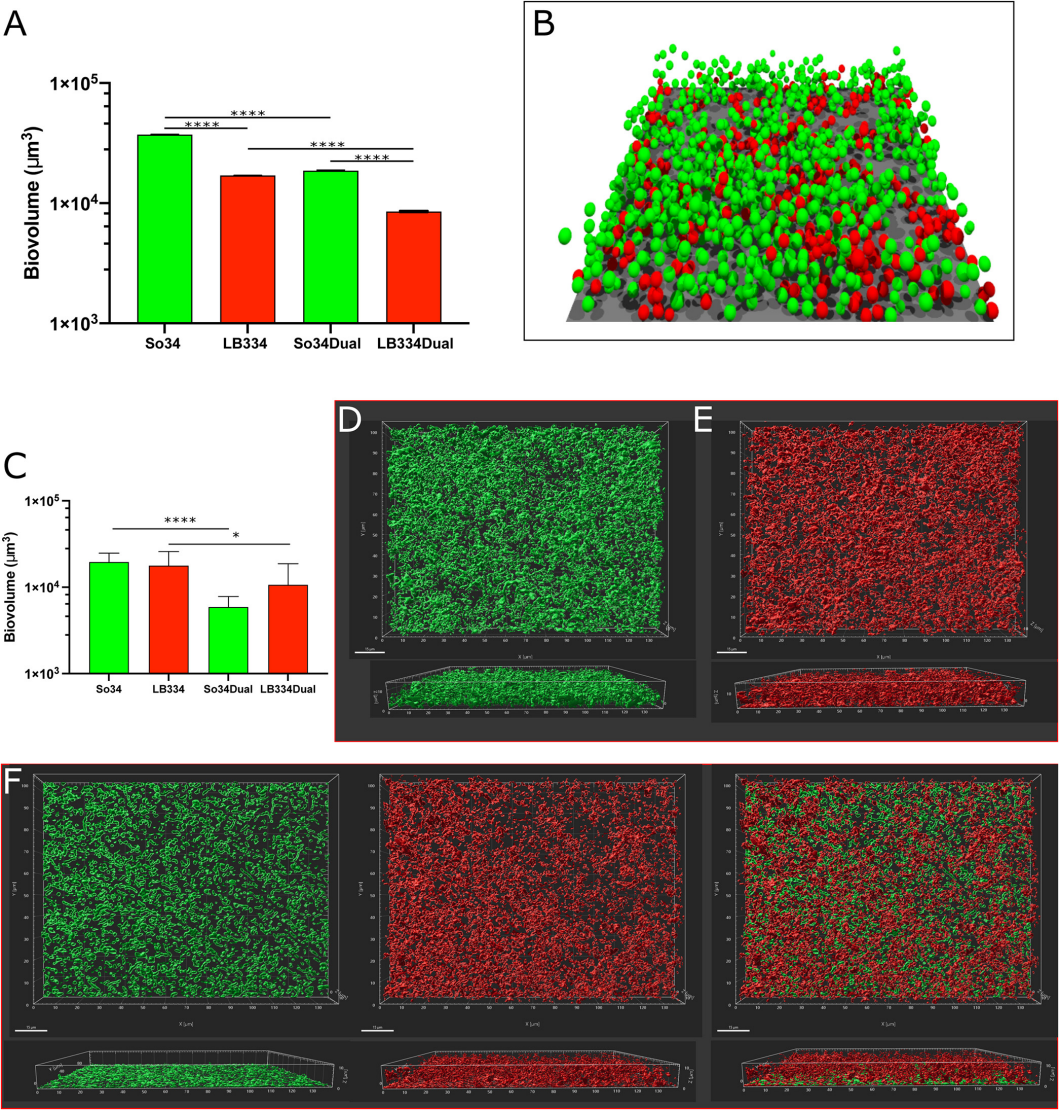
Comparison of simulated and *in vitro* biofilm growth. (A and B) Simulation of biofilm growth in a purely competitive model. (A) Biovolume for single and dual biofilms from simulations of 16-h growth. Results are expressed as mean and standard deviation for 5 simulations with similar initial conditions. LB334, Lactobacillus paracasei; So34, Streptococcus oralis. (B) Final structure of a 16-h dual biofilm simulation. Red spheres, *L. paracasei*; green spheres, S. oralis; extracellular matrix filled the space between cells but is not represented in the image for clarity. The surface area of this image is 18,496 μm^2^. (C to F) *In vitro* biofilm growth. (C) Biovolume plot for 16-h single- and dual-species biofilm cultures. LB334, Lactobacillus paracasei; So34, Streptococcus oralis. Plotted is the average biovolume from 18 microscopic fields, imaged from 8 wells in 4 independent experiments. (D to F) Three-dimensional reconstructions of a 16-h S. oralis mTeal biofilm (D), a 16-h *L. paracasei* (dyed with Cell-Tracker Red) biofilm (E), and a 16-h dual S. oralis*-L. paracasei* biofilm (left, S. oralis; center, *L. paracasei*; right, merged 2-channel image) (F). Reconstructions made in Imaris with Surfaces protocol from 63×, 14-bit images; image dimensions, 139.51 μm (968 pixels) by 104.92 μm (728 pixels). Z-slices = 28 (D), 30 (E), and 23 (F). Bar, 15 μm. ∗, *P* < 0.05; ∗∗∗∗, *P* < 0.0001.

We began by constructing a model where the only interactions between the two species were competition for space and for consumption of nutrients required for growth. Using the iDynoMiCS software (34), we constructed an agent-based model with the two bacterial species competing for glucose and oxygen. The growth parameters of the two species with respect to glucose were initially obtained from the literature (38, 39). However, the biofilm model using these parameter values behaved differently than the experiments; specifically, the simulated biovolume was only weakly affected by medium dilutions, while in the experiments this effect was much stronger (see Fig. S1 in the supplemental material). Thus, we adjusted the μ_max_ and $K_{S_{g}}$ parameters such that the simulation would display as similar dependence on medium concentration as the experiments. The growth parameters of each species with respect to oxygen were taken from the literature (34, 40). Simulations seeded with an initial density of 0.01 cells/μm^2^ of each species, as used in experiments, led to biofilms with properties depicted in Fig. 1A and B.

10.1128/mSphere.00875-21.3 FIG S1 *In vitro* biofilm growth of S. oralis (green) and *L. paracasei* (red) in biofilm medium and medium dilutions from full strength to 0.10× (10% medium, 90% PBS). Biovolume was measured in Imaris software, from images taken after 16 h of growth at 37°C, 5% CO_2_. Lines represent linear regression with 95% confidence limits (dashed lines). Single-species cultures are solid symbols with solid linear regression line, and dual-species cultures are gray-filled symbols with dotted regression lines. Three experiments, 2 biofilms per experiment, 3 to 4 images per biofilm. Linear regression statistics: S. oralis in single-species biofilms, *y* = 10,077*x* + 4,385, *R*^2^ = 0.2838; S. oralis in dual-species biofilms, *y* = −366.2*x* + 4,631, *R*^2^ = 0.003272; *L. paracasei* in single-species biofilms, *y* = 12,367*x* + 13,497, *R*^2^ = −0.3750; *L. paracasei* in dual-species biofilms, *y* = 7,639*x* + 12,717, *R*^2^ = 0.2412. Download FIG S1, EPS file, 0.2 MB. Copyright © 2021 Archambault et al. 2021 Archambault et al. https://creativecommons.org/licenses/by/4.0/ This content is distributed under the terms of the Creative Commons Attribution 4.0 International license.

### Model predictions.

Agent-based models were set up to include each of the species in isolation, as well as a biofilm seeded with equal amounts of the two species. These models were then run to examine how dual biofilms would behave under the hypothesis of simple competition for space and nutrients. As can be seen in Fig. 1A, in single-species biofilms S. oralis is predicted to be a better biofilm former than *L. paracasei*, based on simulation biovolume estimates. As expected, there is a decrease in the biovolume of each species in the dual biofilm compared to the single biofilm, based on nutrient and oxygen competition. Interestingly, based on these parameters, the simulations predicted that in the dual biofilm the biovolume of S. oralis would be higher than that of *L. paracasei* (Fig. 1B).

A question arises whether the competition between the species is happening just for the space their cells occupy or if the carbon substrate is becoming limiting. To check this, we set up a control simulation where the two species have independent carbon sources (substrate 1 and substrate 2) and therefore are competing for only space. The results were very similar to the case in which they both use the same carbon substrate, so we conclude that under the conditions of the original simulation the two species compete for only space. Effectively, the fastest-growing species (S. oralis) controls more space than the slowest (*L. paracasei*). Details of the control simulation can be found in Text S1 in the supplemental material.

10.1128/mSphere.00875-21.1 TEXT S1 Control model and sensitivity analysis: contains information about the control model where the two bacteria compete for space and have two different carbon sources. It also contains the sensitivity analysis for the parameters calibrated for all the four models. Download Text S1, DOCX file, 0.02 MB. Copyright © 2021 Archambault et al. 2021 Archambault et al. https://creativecommons.org/licenses/by/4.0/ This content is distributed under the terms of the Creative Commons Attribution 4.0 International license.

### Live biofilm growth.

We next measured single and dual biofilm growth of S. oralis and *L. paracasei* experimentally to test the model predictions. Single- and dual-species biofilm cultures of S. oralis and *L. paracasei* acidify our biofilm media slightly; at the end of the 16-h growth period, the starting pH of 8 was reduced to pH 6.5. Each species attained a similar biovolume when grown alone for 16 h, in contrast to the simulation, which predicted a lower biovolume for *L. paracasei*. In coculture, *L. paracasei* growth was predictably slightly lower than in single culture, and S. oralis biovolume was significantly reduced (Fig. 1C). This was different from the simulation, which predicted similar reductions in growth for the two species. Three-dimensional (3D) projections of biovolume made from images of single and dual biofilms illustrate the altered growth pattern of S. oralis in biofilms with *L. paracasei* (Fig. 1D to F). When growing alone, S. oralis biofilm takes the form of interconnecting mounds of cells and reaches thicknesses of 10 to 14 μm (Fig. 1D). When growing with *L. paracasei*, S. oralis grows to a height of only 3 to 5 μm (Fig. 1F, left image). *L. paracasei* maintains a similar growth pattern in single- and dual-species biofilms, a dense layer of cells approximately 8 to 10 μm thick (Fig. 1E and F, middle image). Because the 2 species formed biofilms with different configurations, we suspected there could be an imperfect correlation between biovolumes and cell counts. We therefore used genus- or strain-specific real-time quantitative PCR (qPCR) of 16S rRNA to estimate the number of each bacterial species that grew in the biofilms (Fig. S2B). The results of qPCR indicated that single-species biofilms of S. oralis contained more cells than single-species *L. paracasei* biofilms as predicted by the model. However, in dual-species biofilms, the number of S. oralis cells was significantly lower than in the biofilms containing S. oralis alone, and this contrasted markedly with the model predictions. Overall, these experiments indicated that actual biofilm growth did not match the predictions of the first iteration of our model in which competitions for nutrients and space were the only interactions between the 2 species.

10.1128/mSphere.00875-21.4 FIG S2 (A) Number of bacterial cells (agents) for single and dual biofilms from simulations of 16-hour growth of a purely competitive model. Results are expressed as mean and standard deviation for 5 simulations with similar initial conditions. LB334, Lactobacillus paracasei; So34, Streptococcus oralis. (B) Cell numbers in *in vitro* biofilms grown for 16 h at 37°C, 5% CO_2_, as measured by qPCR targeting 16S rRNA gene for each species. Data are from 3 experiments, *n* = 6 biofilms of each type: S. oralis alone, *L. paracasei* alone, and dual-species biofilm. Download FIG S2, EPS file, 0.07 MB. Copyright © 2021 Archambault et al. 2021 Archambault et al. https://creativecommons.org/licenses/by/4.0/ This content is distributed under the terms of the Creative Commons Attribution 4.0 International license.

### Exploration of the possible interactions within the biofilm.

Since we observed experimentally that there is a dramatic reduction in biovolume of S. oralis when it is grown with *L. paracasei* compared to when it is grown alone, we proposed two plausible explanations for these interactions:

1. *L. paracasei* product(s) may inhibit growth of S. oralis cells (41, 42), or
2. *L. paracasei* may secrete a surfactant (27–29) that causes both cell types to detach from the biofilm.

In the model, the growth inhibition mechanism was incorporated by including the production of a bacteriostatic product by *L. paracasei* and its effect on slowing the growth of S. oralis. To include a surfactant mechanism in the simulations, we used a modified version of iDynoMiCS (36) in a way that allowed the biofilm cells of both bacteria to disperse from the biofilm surface based on the local surfactant concentration (see Materials and Methods). We also ran a model which incorporated both mechanisms. The simulations were repeated multiple times for the single and dual biofilm in all three models. A sufficient number of repetitions was run so as to achieve a coefficient of variation smaller than 10%. For the inhibition model, it was 5 runs, and for the models containing surfactant, it was 50.

**FIG 2:**
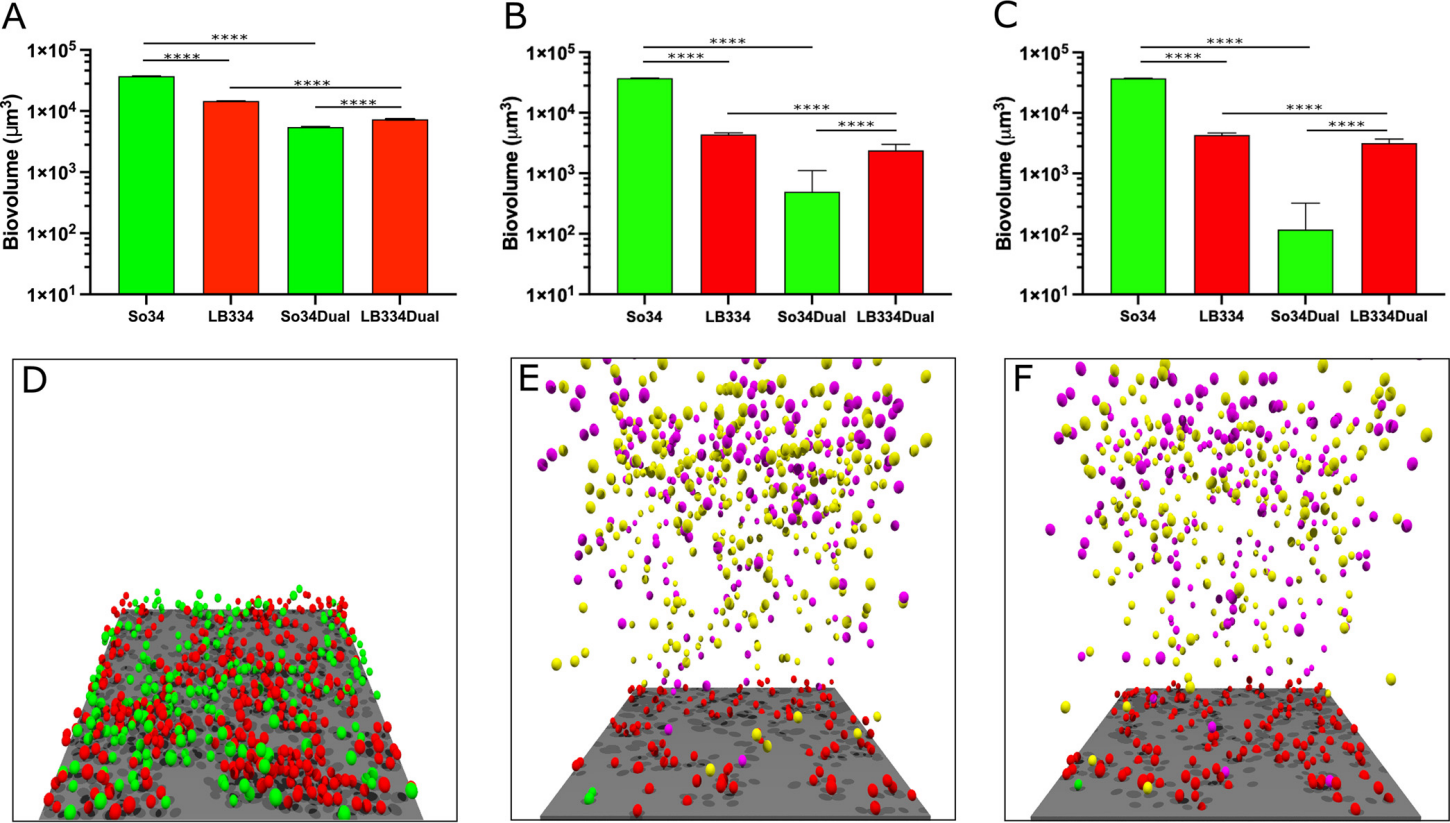
Simulation of biofilm growth in an inhibition, surfactant, and inhibition + surfactant model. (A) Biovolume plot for single and dual biofilms from the simulation of a 16-h biofilm with growth inhibition. LB334, Lactobacillus paracasei; So34, Streptococcus oralis. Results are expressed as mean and standard deviation for 5 simulations with the same initial conditions as the competition model. (B) Biovolume plot for single and dual biofilms from the simulation of a 16-h biofilm with surfactants. The model was repeated 50 times to get a low coefficient of variation. (C) Biovolume plot for single and dual biofilms from the simulation of 16-h biofilm with inhibition and surfactants. The model was repeated 50 times to get a low coefficient of variation. (D) Final structure of a 16-h dual inhibition model simulation. Red spheres, *L. paracasei*; green spheres, S. oralis; extracellular matrix filled the space between cells but is not represented in the image for consistency with surfactant images. The image with EPS in the inhibition model is available in the supplemental material (Fig. S5). The surface area of this image is 18,496 μm^2^. (E) Final structure of a 16-h dual surfactant model. The magenta and yellow spheres floating above the biofilm are the planktonic cells of *L. paracasei* and S. oralis, respectively. (F) Final structure of a 16-h dual model with inhibition and surfactants. ∗∗∗∗, *P* < 0.0001.

In Fig. 2, the growth inhibition model (Fig. 2A), the surfactant model (Fig. 2B), and their combination (Fig. 2C) show that the biovolume of S. oralis is lower than *L. paracasei* in the dual biofilm, suggesting that either or both mechanisms could be responsible for the decrease in S. oralis biovolume in biofilms with *L. paracasei*. In the combination model, we see that the S. oralis cells nearly all detach from the biofilm, and thus, its biovolume in the dual biofilm is close to zero. Figures 2D to F show the images of the simulated dual biofilm at 16 h after inoculation. The images in Fig. 2E and F, depicting the simulation of the models with surfactant production, also show the floating planktonic cells that had detached from the periphery of the biofilm. The planktonic cells of S. oralis and *L. paracasei* are a different color to distinguish the planktonic from the biofilm cells.

10.1128/mSphere.00875-21.6 FIG S4 S. oralis single-species biofilms are thick and lush, but S. oralis reaches only a few micrometers of thickness in dual-species biofilms with *L. paracasei*. (A to D) Sectioned 63×, 14-bit images of S. oralis (A and C) and *L. paracasei* (B and D) single-species (A and B) and dual-species (C and D) biofilms grown for 16 h at 37°C, 5% CO_2_. Within each image group, the large rectangular image shows the second Z-slice, 1 μm above the base of the biofilm. The thin images at the right and bottom are side views through all Z-slices at the location of the white lines on the large image. Image dimensions: 139.51 μm (968 pixels) by 104.92 μm (728 pixels). Number of 0.5-μm-thick Z-slices = 28 (B), 30 (C), and 23 (D). Bar, 15 μm. (E) This is a vertical profile of the simulation of the 2-species biofilm where they compete for glucose, oxygen, and space. It shows that S. oralis outgrows *L. paracasei* in the competition-only model. Download FIG S4, EPS file, 0.5 MB. Copyright © 2021 Archambault et al. 2021 Archambault et al. https://creativecommons.org/licenses/by/4.0/ This content is distributed under the terms of the Creative Commons Attribution 4.0 International license.

10.1128/mSphere.00875-21.7 FIG S5 Simulation of biofilm growth in an inhibition, surfactant, and inhibition + surfactant model. (A) Biovolume plot for single and dual biofilms from the simulation of a 16-hour biofilm with growth inhibition. LB334, Lactobacillus paracasei; So34, Streptococcus oralis. Results are expressed as mean and standard deviation from 5 simulations with the same initial conditions as the competition model. (B) Biovolume plot for single and dual biofilms from the simulation of a 16-hour biofilm with surfactants. The model was repeated 50 times to get a low coefficient of variation. (C) Biovolume plot for single and dual biofilms from the simulation of 16-hour biofilm with inhibition and surfactants. The model was repeated 50 times to get a low coefficient of variation. (D) Final structure of a 16-hour dual inhibition model simulation. Red spheres, *L. paracasei*; green spheres, S. oralis; EPS is denoted by black (for S. oralis) and gray (for *L. paracasei)* spheres. The surface area of this image is 18,496 μm^2^. (E) Final structure of a 16-hour dual surfactant model. The magenta and yellow spheres floating above the biofilm are the planktonic cells of *L. paracasei* and S. oralis, respectively. (F) Final structure of a 16-hour dual model with inhibition and surfactants. Download FIG S5, EPS file, 1.6 MB. Copyright © 2021 Archambault et al. 2021 Archambault et al. https://creativecommons.org/licenses/by/4.0/ This content is distributed under the terms of the Creative Commons Attribution 4.0 International license.

Because the simulations of the two hypotheses resulted in somewhat similar outcomes (at least qualitatively), we are not able to eliminate either of them as possible explanations of how S. oralis is affected by *L. paracasei* in mixed biofilms. Thus, we looked for experimental evidence for the action of either an inhibitory substance, a surfactant, or perhaps both. Our culture wells contained both attached cells (in the biofilm) and planktonic cells (removed with spent medium before imaging), and we reasoned that a surfactant or an inhibitory substance might alter the number of planktonic and biofilm cells in different ways. We expected that a toxin released into the surrounding medium could reduce the number of cells in both the biofilm and the planktonic phase while a surfactant would tend to increase the proportion of planktonic bacteria without necessarily affecting the overall number of cells in each culture well. To determine the number of cells in each phase, we enumerated each species using qPCR with genus (for *Lactobacillus*)- or strain (for Streptococcus)-specific primers.

**FIG 3:**
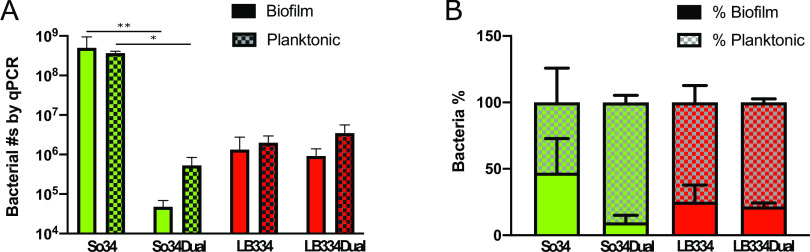
Experimental data demonstrate inhibition of S. oralis biofilm formation by *L. paracasei*. (A) Plot of bacterial numbers in biofilm (solid bars) and planktonic state (checkered bars) for single-species and dual-species biofilms determined by qPCR. Average of bacterial numbers from 4 biofilms. LB334, *L. paracasei*; So34, S. oralis. ∗, *P* < 0.05; ∗∗, *P* < 0.01. (B) Data from panel A plotted as a percentage of the total bacteria per well.

The total number of S. oralis cells in the biofilm, as counted by qPCR, was significantly reduced in the dual-species biofilms (Fig. S2B). We determined that the number of planktonic S. oralis cells was also significantly reduced when *L. paracasei* was present (Fig. 3A). *Lactobacillus* numbers were similar for biofilms and for planktonic growth in single- and dual-species cultures by this quantification method (Fig. 3A). Because the number of planktonic S. oralis cells was reduced along with the biofilm cells, we suspected a growth-inhibitory toxin was present.

We reasoned that a surfactant might increase the percentage of cells that detached from the biofilm, regardless of the overall growth of the bacteria in the well. To determine whether this occurred, the same qPCR data were plotted as a percentage of the total number of cells in each well (Fig. 3B). Interestingly, the percentage of S. oralis planktonic cells was higher in the mixed cultures than in the single cultures, while *L. paracasei* appeared to be unaffected, showing similar percentages of planktonic cells in single and dual cultures. Considering these results, the data suggest that the presence of surfactant activity is additionally plausible, indicating that more than one mechanism may be involved in the suppression of S. oralis biofilm growth in the presence of *L. paracasei*.

### Further experimental exploration of the system.

**FIG 4:**
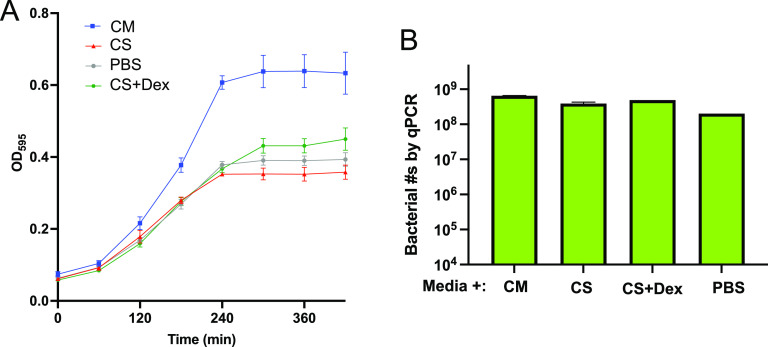
Supernatants from *L. paracasei* biofilm cultures reduce growth of S. oralis. Cell-free supernatants were prepared from 24-h cultures of *L. paracasei* biofilms and filtered to concentrate molecules of >3 kDa in molecular weight. (A) S. oralis planktonic cultures were grown in a 1:1 mixture of media (RPMI/BHI/FBS) and one of the following: CM (concentrated medium prepared by filtering by the same method as for *L. paracasei* concentrated supernatant) (blue symbols), CS (concentrated supernatant) (red symbols), PBS (gray symbols), and CS + Dex (concentrated supernatant supplemented with 1.8% dextrose to compensate for depletion of carbon/energy source by *L. paracasei*) (green symbols). (B) S. oralis biofilms were grown in the mixtures described for the left panel, above, for 16 h, and bacterial numbers in biofilms were determined by qPCR.

To further explore *L. paracasei* activity against S. oralis, we grew S. oralis in medium mixed with concentrated, cell-free supernatants from *L. paracasei* biofilms. The cell-free supernatants were concentrated to approximately one-fourth their original volume using centrifugal filter units which limited the molecular weight of molecules in the concentrates to greater than 3-kDa nominal molecular weight. We first tested the ability of S. oralis to grow in planktonic culture containing a 1:1 mixture of biofilm medium plus either concentrated *L. paracasei* spent supernatant, concentrated medium, phosphate-buffered saline (PBS), or concentrated supernatant plus 1.8% dextrose to compensate for the depletion of this carbon/energy source by *L. paracasei*. The supernatants isolated from 24-h *L. paracasei* biofilm cultures, and the concentrated supernatant made from them, had a pH of 6.0, while the concentrated medium control had a pH of 8. Due to the buffering capacity of the fresh medium used to dilute the supernatants, pH of all treatments was 7.5 to 8 at the start of incubation. Growth of S. oralis was limited by *L. paracasei* supernatant to slightly more than one-half the level reached in the concentrated medium control (Fig. 4A). As might be expected, a control containing PBS limited growth to about the same degree as the supernatant, but adding back a carbon/energy source (1.8% dextrose) rescued S. oralis growth to only a small degree.

We next tested the effect of concentrated *L. paracasei* supernatant on biofilm growth of S. oralis. Biofilms were seeded for 1 h in medium and then were switched to a 1:1 mixture of biofilm medium plus either concentrated *L. paracasei* spent supernatant, concentrated medium, PBS, or concentrated supernatant plus 1.8% dextrose. *L. paracasei* concentrated supernatant had a minimal negative effect on the quantity of S. oralis accumulated in biofilms after 16 h of growth, as measured by qPCR (Fig. 4B). We conclude that, in spite of its ability to suppress S. oralis growth in planktonic culture, concentrated *L. paracasei* supernatant is not sufficient to explain the suppression of S. oralis biofilm growth seen in live cocultures (Fig. 4A).

Lactic acid bacteria, including *L. paracasei*, are known to produce peptides and proteins with antimicrobial properties collectively known as bacteriocins (43–45). To explore the possibility of cytotoxic activity by *L. paracasei*, we treated preformed 16-h S. oralis biofilms with the filtered, concentrated, cell-free supernatants from *L. paracasei* biofilms for 22 h. We then used a live/dead stain and fluorescence imaging to determine the viability of S. oralis in these biofilms (Fig. S3). The proportions of live cells were not significantly different in biofilms treated with filtrate containing molecules smaller than 3 kDa, unconcentrated medium, or concentrated medium controls (Fig. S3A). Rhamnolipid surfactants from Pseudomonas aeruginosa are known to have antimicrobial properties (46). Commercially prepared rhamnolipids at 100 μg/ml and 200 μg/ml increased the proportion of nonviable cells in a dose-dependent (although not statistically significant) way (Fig. S3A). Concentrated medium from *L. paracasei* biofilms containing molecules larger than 3 kDa significantly reduced the proportion of viable cells in S. oralis biofilms (Fig. S3A). These results suggest that one or more high-molecular-weight products secreted by *L. paracasei* could act as bacteriocins against S. oralis in mixed-species biofilms.

10.1128/mSphere.00875-21.5 FIG S3 Supernatant from *L. paracasei* biofilm cultures is bactericidal against S. oralis in established biofilms. (A) Number of live (green) and dead (red) S. oralis cells in biofilms exposed to CM (concentrated medium control) (*n* = 15), UCS (unconcentrated supernatant) (*n* = 8), FIL (filtrate) (*n* = 13), R100 (rhamnolipid positive control, 100 μg/ml) (*n* = 16), R200 (200 μg/ml) (*n* = 8), HS (half-strength concentrated supernatant) (*n* = 17), or FS (full-strength concentrated supernatant) (*n* = 15). Data from two experiments; *n* denotes the number of images analyzed. (B) Representative 63×, 14-bit images of live/dead staining of S. oralis cells in biofilms exposed to each condition. Shown are 3D projections viewed from above. Image dimensions: 36.9 μm (256 pixels) square. The number of 0.5-μm-thick Z-slices was adjusted according to the thickness of the biofilms: Z = 38 (CM), 38 (UCS), 40 (FIL), 48 (R100), 54 (R200), 47 (HS), and 32 (FS). Bar, 5 μm. Download FIG S3, EPS file, 2.4 MB. Copyright © 2021 Archambault et al. 2021 Archambault et al. https://creativecommons.org/licenses/by/4.0/ This content is distributed under the terms of the Creative Commons Attribution 4.0 International license.

## DISCUSSION

We have used a combined modeling and experimental process to construct and refine a model of dual-species biofilm growth which has, in turn, informed further experimental exploration of interspecies interactions in biofilms. Our initial agent-based model was limited to nutrient and space competition and did not fully explain the interaction between S. oralis and *L. paracasei.* To account for the differences between model and experimental results, we proposed two hypotheses for the inhibition of S. oralis biofilm growth: an inhibitory substance or a surfactant produced by *L. paracasei*. These hypotheses were translated into three different models, and simulations were carried out. In all three cases, the biovolume and cell numbers of S. oralis were reduced in the dual biofilm simulations compared to the single biofilm simulations, indicating that the experimental results could have been caused by either mechanism or both. Differences in S. oralis planktonic and biofilm growth in single- and dual-species biofilms *in vitro* also supported both mechanisms. We further explored the interaction experimentally and found that concentrated supernatants from *L. paracasei* biofilm cultures contained a substance which reduced S. oralis planktonic growth and was toxic to cells in S. oralis biofilms. Prior to this study, *L. paracasei* and S. oralis interactions in biofilms had not been studied experimentally, and this is one of a few studies using a combination of agent-based models and experiments to study interspecies biofilm interactions.

Members of the viridans group Streptococcus spp. have putative pathogenic roles, contrasted with the probiotic properties of *Lactobacillus* spp., and interactions between members of these genera have long been of interest to the medical community. For example, many *Lactobacillus* strains and their cell-free supernatants have antimicrobial activity against streptococcal pathogens including S. mutans (23–25, 47) and S. pyogenes (26, 48). With a few notable exceptions (25), the mechanisms of inhibition are not fully understood. Interactions between Streptococcus and *Lactobacillus* are often species specific, and while most examples in the literature involve *Lactobacillus* inhibition of Streptococcus spp., at least one example of the reverse has been found (49).

Surfactants are amphiphilic molecules that reduce surface tension and interfacial tension, thus interfering with adhesive interactions between microbes and the surfaces to which they attempt to attach. Biosurfactants produced by *Lactobacillus* spp. have been explored as a means of inhibiting biofouling in commercial applications and in prevention of biofilms growing on hard surfaces in the oral environment (22, 28, 29). Many studies have found biosurfactant activity in supernatants of *Lactobacillus* culture (18, 50–52). Many of the mixtures and molecules isolated from supernatants of *Lactobacillus* spp. have both antimicrobial and biosurfactant activities, consistent with our findings using concentrated spent medium (22, 27–29, 53).

Streptococcus and *Lactobacillus* species are known to be both acidogenic and acid tolerant. As we reported, acidification occurs in our single- and dual-species biofilm cultures. However, the buffering capacity of the medium keeps the pH well above the lower limit for exponential growth of S. oralis: pH 4.14 to 4.88 for 12 Streptococcus species and strains, including S. oralis (54). Lower pH levels of 3.0 to 3.5 are required for killing of S. oralis (54). Therefore, it is unlikely that the pH of the culture medium is responsible for the reported growth inhibition and death of S. oralis in dual-species cultures and when treated with *L. paracasei* spent medium preparations.

Our experimental results support the presence of a substance with growth inhibition and antimicrobial properties in concentrated supernatants from *L. paracasei* biofilm cultures, while enumeration of planktonic and biofilm-associated cells hints at a mechanism that affected adhesion or dispersal of S. oralis. While the concentrated supernatant reduced growth of planktonic cultures monitored over 7 h, it did not change the final cell numbers of S. oralis biofilms measured after 16 h of growth. This could be the result of the longer incubation time and constraints on the maximum size of S. oralis biofilms not conducive to detachment/dispersion, but also differences in growth rates between planktonic and biofilm growth forms. It is also possible that our concentrated cell-free *Lactobacillus* spent medium contains more than one active antistreptococcal compound. Further fractionation and purification of *L. paracasei* supernatants could lead to identification of the substance or substances responsible for the effects we observed on S. oralis biofilms.

Our agent-based model based in iDynoMiCS (34) is one of very few models which have been constructed with constant interaction and feedback with experimental work (36, 37). The growth parameters in the model were based on the growth of single-species biofilms in different concentrations of the medium, and prior published values. We investigated further interactions, namely, noncompetitive inhibition and surfactant effects, based on the observed interactions of S. oralis and *L. paracasei in vitro*.

The surfactant model was constructed using a modified version of iDynoMiCS (36) which incorporated the transition of biofilm cells to planktonic cells based on the local surfactant concentration. A limitation of the model was that the area considered was much smaller than that used in experiments, due to limitations on computational resources. However, the ratio of initial number of seed cells to surface area was the same in the experiments and simulations. Effectively, the simulations represent a small section of the biofilms grown experimentally but have similar characteristics in terms of biofilm thickness.

Interkingdom interactions such as those between oral bacteria and the fungus C. albicans hold great interest due to medical treatments or immunocompromised states resulting in fungal-bacterial dysbiosis, a contributing factor in human disease (55). In certain host backgrounds S. oralis synergizes with C. albicans to increase the virulence of the fungus (5, 56–58). *L. paracasei*, on the other hand, inhibits the transition of C. albicans yeast to hyphal form (23). Our next goal is to increase the complexity of our experimental and mathematical models by incorporating C. albicans to study interactions in three-species biofilms. An assumption made in this agent-based model is that all the cells (agents) are spherical in shape. One avenue of future research will be to incorporate the rod shape (for lactobacilli) and filamentous shape (for fungi) into the software and observe the effect, if any, that these cell morphologies may have on the biofilm structure and dynamics. The insights gained through the iterative process of modeling and experimentation in our study of S. oralis and *L. paracasei* interactions during biofilm growth will guide us in exploring more complex, multispecies biofilms.

## MATERIALS AND METHODS

### Bacterial strains and culture methods.

Fluorescent S. oralis 34 teal (So34) (59, 60) from glycerol stocks was grown overnight in brain heart infusion (BHI) medium (Becton, Dickinson and Company, Sparks, MD, USA) supplemented with erythromycin, 5 μg/ml, as needed, under aerobic static conditions at 37°C with 5% CO_2_. *L. paracasei* ATCC 334 (LB334) was similarly cultured in De Man, Rogosa, and Sharpe (MRS) broth. Subcultures were grown from overnight cultures to an optical density reading at 600 nm (OD_600_ reading) of 1.0 (1 × 10^8^ cells/ml). Prior to biofilm growth, LB334 bacteria were labeled with Cell-Tracker Red CMTPX dye (Invitrogen, Carlsbad, CA, USA) according to the manufacturer’s protocol. Briefly, cells were diluted 1:10 in PBS, centrifuged 5 min at 2,000 × *g*, resuspended at a density of 1 × 10^7^ to 1 × 10^8^ cells/ml in 18 μM dye in PBS, and then incubated 45 min at 37°C. The labeled cells were centrifuged, washed once in PBS, and resuspended in biofilm growth medium. So34 cultures were diluted 1:10 in PBS. To satisfy the nutritional requirements of both bacterial species, biofilms were grown in an optimized complex medium containing 80% RPMI (Roswell Park Memorial Institute) 1640 without l-glutamine, without phenol red (ThermoFisher Scientific, Waltham, MA, USA), 10% BHI (BBL brain heart infusion; Becton, Dickinson and Company, Sparks, MD, USA), and 10% fetal bovine serum (FBS; R&D Systems, Minneapolis, MN, USA) (5). Bacteria at 1 × 10^6^ were seeded into μ-Slide 8-well chambered coverslips (Ibidi GmbH, Gräfelfing, Germany) and incubated at 37°C with 5% CO_2_ for 1 h, the supernatant containing unattached bacteria was removed and replaced with fresh medium, and incubation continued for 16 or 24 h. Prior to imaging, media were removed and replaced with PBS.

### Microscopy and image analysis.

Biofilms were imaged on a Zeiss Axio Observer inverted microscope with Apotome2 (Carl Zeiss, Inc., Thornwood, NY, USA). Unless otherwise noted, images were made using a 63× oil immersion lens. Images were analyzed using Imaris software (Oxford Instruments plc, Abingdon, Oxon, UK). Biovolume of biofilms was measured from 3D reconstructions using the “surfaces” protocol, and live and dead cells were counted using the “spots” protocol in Imaris. Briefly, to measure biovolumes, we manually set a threshold intensity which excluded background fluorescence and then created “surfaces” which represent the volume occupied by each bacterial species. Objects less than ∼1 μm^3^ were excluded, and the output volumes were added to obtain the total biovolume per image. To count individual live and dead cells from images, the diameter of spots (cells) was estimated to be 0.8 μm, background subtraction was applied to the images, and the total number of spots was recorded.

### qPCR enumeration of biofilms.

Biofilms were grown as described above. Supernatants were removed at 1 h and 16 h, placed in sterile 2-ml tubes, and centrifuged 5 min at 10,000 × *g*, and pellets were frozen at −80°C. Biofilm starting cultures (1 h) and mature (16 h) biofilms were frozen in their imaging well slides immediately after imaging. DNA extraction was carried out using the DNeasy blood and tissue kit (Qiagen, Germantown, MD, USA) according to manufacturer’s instructions, including the suggested pretreatment with enzymatic lysis buffer for Gram-positive bacteria. Genomic DNA was eluted in 100 μl nuclease-free water. qPCR was carried out using S. oralis 34 strain-specific primers (*wefA-wefH*, forward, 5′-CATCAAGAACTTCTCGGAGTTG-3′; reverse, 5′-CCACAGCTCCAGAATATTTAGC-3′) (57) and All Lacto primers (forward, 5′-TGGATGCCTTGGCACTAGGA-3′; reverse, 5′-AAATCTCCGGATCAAAGCTTACTTAT-3′) (61).

### Concentrated supernatants.

LB334 biofilms were grown in polystyrene 6-well plates for 24 h under static conditions at 37°C with 5% CO_2_. Supernatants were centrifuged at 2,200 × *g* for 10 min and then filtered (0.2-μm pore size). The supernatant was concentrated by centrifugation in Centriprep centrifugal filters, 3,000 nominal molecular weight limit (NMWL) (EMD Millipore, Billerica, MA, USA) for 2 spins of 30 min and then one of 10 min, all at 3,000 × *g*. Filtered but unconcentrated supernatant, concentrated supernatant, and filtrate were frozen at −20°C. Unconditioned medium was also concentrated and used as a control. S. oralis planktonic cultures were grown in 96-well plates in a 1:1 mixture of medium (RPMI/BHI/FBS) and either concentrated unconditioned medium, concentrated supernatant, PBS (gray symbols), or concentrated supernatant supplemented with 1.8% dextrose. S. oralis bacteria were allowed to attach for 1 h in RPMI/BHI/FBS medium and then were grown in the supernatant and control mixtures described above for 16 h at 32°C and 5% CO_2_. Bacterial numbers in biofilms were determined by qPCR as described above.

### Data analysis and statistics.

The biovolume and qPCR data were graphed, and statistical analyses were carried out in Prism9 (GraphPad Software, San Diego, CA, USA). Live biofilm data were analyzed by ordinary one-way analysis of variance (ANOVA) with Sidak’s multiple-comparison test.

### Agent-based model and simulations.

All simulations shown were carried out with the iDynoMiCS software (34), which can run agent-based simulations of biofilms including multiple species. The models in this paper include two bacterial species, S. oralis and *L. paracasei*, which are represented by two different types of agents in iDynoMiCS. These agents are governed by rules that represent cell growth, cell division, cell death, production of extracellular polymeric substances (EPS), cell shoving, and cell detachment from the biofilm (34). The full ODD (overview, design, details) protocol according to the work of Grimm et al. (62) for all the agent-based models is included in Text S2 in the supplemental material. The software also simulates medium nutrient diffusion and a liquid phase above the biofilm. Postprocessing of simulation results was carried out with R (63) scripts, and images were rendered with the PovRay (64) software. All the relevant code and the iDynoMiCS protocol files for the different models can be obtained from https://github.com/skoshyc/StrepLactoBiofilmModeling.

10.1128/mSphere.00875-21.2 TEXT S2 ODD (overview, design, details) protocol according to the work of Grimm et al. (V. Grimm, U. Berger, D. L. DeAngelis, J. G. Polhill, et al., Ecol Modell 221:2760–2768, 2010, https://doi.org/10.1016/j.ecolmodel.2010.08.019) for all the agent-based models. Download Text S2, DOCX file, 0.07 MB. Copyright © 2021 Archambault et al. 2021 Archambault et al. https://creativecommons.org/licenses/by/4.0/ This content is distributed under the terms of the Creative Commons Attribution 4.0 International license.

The models presented here are three-dimensional, with a computational grid of 136 by 136 by 136 μm in size. Only parameters related to growth, surfactant and toxin production, the inhibitory constant, and tolerance to surfactant were calibrated. A full sensitivity analysis was carried out for these parameters and is included in Text S1. All the other parameters were kept at default iDynoMiCS values (34).

The Monod growth rate for each cell size is given by:

$$\frac{\mathrm{dX}}{\mathrm{dt}}\;=\;\mu_{\text{max}}\frac{S_{g}}{K_{S_{g}}\;+\;S_{g}}\frac{S_{o}}{K_{S_{o}}\;+\;S_{o}}X$$

where *X* is the biomass of the cell. The two nutrients considered here are glucose and oxygen, and their concentrations are denoted by *S_g_* and *S_o_*, respectively. μ_max_ is the maximum specific growth rate with unit 1/h, and *K_S_* (unit of grams/liter) is the value of the substrate *S* when the specific growth rate is μ_max_/2.

### Estimation of growth parameters.

The growth parameters specific to glucose (namely, μ_max_ and $K_{S_{g}}$) obtained from the literature were for the biofilm growth of Streptococcus gordonii (39) and the growth of Lactobacillus casei in a bioreactor (38). Using these literature parameter values, we then ran 16-h simulations of single-species biofilms for different glucose concentrations (2 g/liter, 1.5 g/liter, 1 g/liter, 0.5 g/liter, 0.2 g/liter, 0.1 g/liter, 0.02 g/liter). The initial value of 2 g/liter was set to match the sugar present in the biofilm medium used in our experiments. On running simulations for different glucose concentrations, it was observed that the biovolume of the species S. oralis and *L. paracasei* did not reduce with reduction in glucose, in contrast to what was observed in biofilm experiments (Fig. S1). We assumed a biologically relevant range of the glucose growth parameters (namely, μ_max_ and $K_{S_{g}}$). We then optimized the parameters within that range (one at a time using the bisection method; see, e.g., reference 65) such as to obtain similar reductions in biovolume upon reducing carbon source similarly to our experiments. The parameter values which gave rise to biovolume reduction with respect to glucose dilution are the ones listed in Table 1. The parameter values with respect to oxygen (i.e., $K_{S_{o}}$) for each species were as in the literature (34, 40). The final parameter values used in all the simulations are displayed in Table 1.

**TABLE 1:** Parameter values used for cell growth of the two species in the biofilm simulations

Parameter	S. oralis	*L. paracasei*
Initial glucose concn *S_g_* (g/liter)	2	2
Initial oxygen concn *S_o_* (g/liter)	0.0064	0.0064
μ_max_ (1/h) for growth	0.32	0.153
*K_Sg_* (g/liter)	1.756	1.2
*K_So_* (g/liter)	0.192e−3	0.2e−3
*K_I_* (g/liter)	0.0025	
Yield_glucose	−3	−0.17
Yield_oxygen	−2	−1
Biomass/capsule ratio	0.8:0.2	0.9:0.1
*k* (1/h) of production of toxin and surfactant by *L. paracasei*		0.7
Yield of inhibitor by *L. paracasei* (g/g)		0.3
Yield of surfactant by *L. paracasei* (g/g)		0.4

All biofilm simulations were started with a seed of 176 cells. In the case of mixed-species biofilm simulations, there were 88 cells of each type, such that the same total number of cells is kept constant. This matches the experiments which seeded the biofilms at 0.01 cells/μm^2^.

### Noncompetitive inhibition.

In this case the simulation includes secretion by *L. paracasei* of an inhibitor of the growth of S. oralis through noncompetitive kinetics. This required including the term *K_I_*/*K_I_* + *I* in the growth rate equation of S. oralis:

$$\frac{\mathrm{dX}}{\mathrm{dt}}\;=\;\mu_{\text{max}}\frac{S_{g}}{K_{S_{g}}\;+\;S_{g}}\frac{S_{o}}{K_{S_{o}}\;+\;S_{o}}\frac{K_{I}}{K_{I}\;+\;I}X$$

where *K_I_* is the concentration of the inhibitory substance *I* that gives rise to a specific growth rate of μ_max_/2.

**FIG 5:**
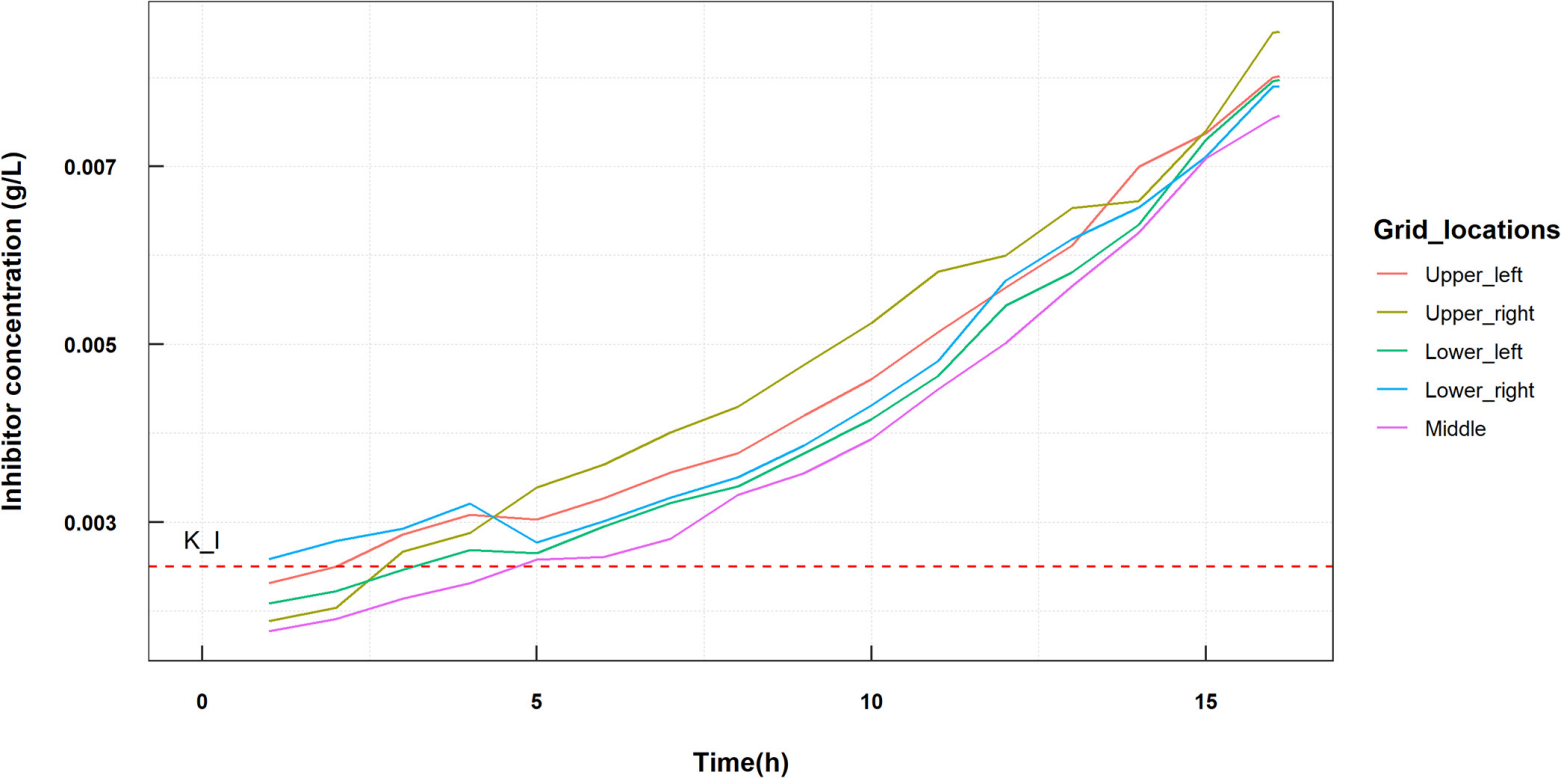
Inhibitor concentration in a dual biofilm at different time points in the simulation. Data were sampled at different locations of the biofilm, the four corners and the center of the surface area covered by the simulation. The red dashed line is the value of *K_I_* = 0.0025, which shows that the inhibitory effect of the toxin on S. oralis is present from around 5 h.

Since this is a hypothetical inhibitor, we cannot match its parameters to any real data. What is important is to have the *K_I_* for S. oralis to be around the concentration of that substance in the biofilm, so as to effectively cause an inhibition (Fig. 5). The inhibitor production is given as a separate first-order reaction with *k* = 0.7 (1/h) with a yield of 0.3 of the inhibitor. We set *K_I_* arbitrarily to 0.0025.

### Surfactant simulation.

To simulate the action of a surfactant, we utilized the software from reference 36 that is a modified version of iDynoMiCS 1.1 software. The original intent of the authors’ modification was to include secretion of a chemotaxis agent (36). In that modification the chemotaxis agent repels Helicobacter pylori cells which detach from the biofilm. For our purposes, the surfactant produced by *L. paracasei* plays a similar role as that repellant; however, the effect is now on both cell types because the action of the surfactant is of a physicochemical nature affecting both cell types (though not necessarily to the same degree, since that could be determined by their cell wall composition). The surfactant is produced by *L. paracasei* cells, and when its concentration becomes higher than a threshold, it causes cells of both species to detach from the biofilm and become planktonic. Any cell in the periphery of the biofilm is subject to detachment based on the concentration of surfactant in its vicinity (36). The planktonic cells are in the boundary layer right below the bulk medium (36). In the model, EPS production is not included.

**FIG 6:**
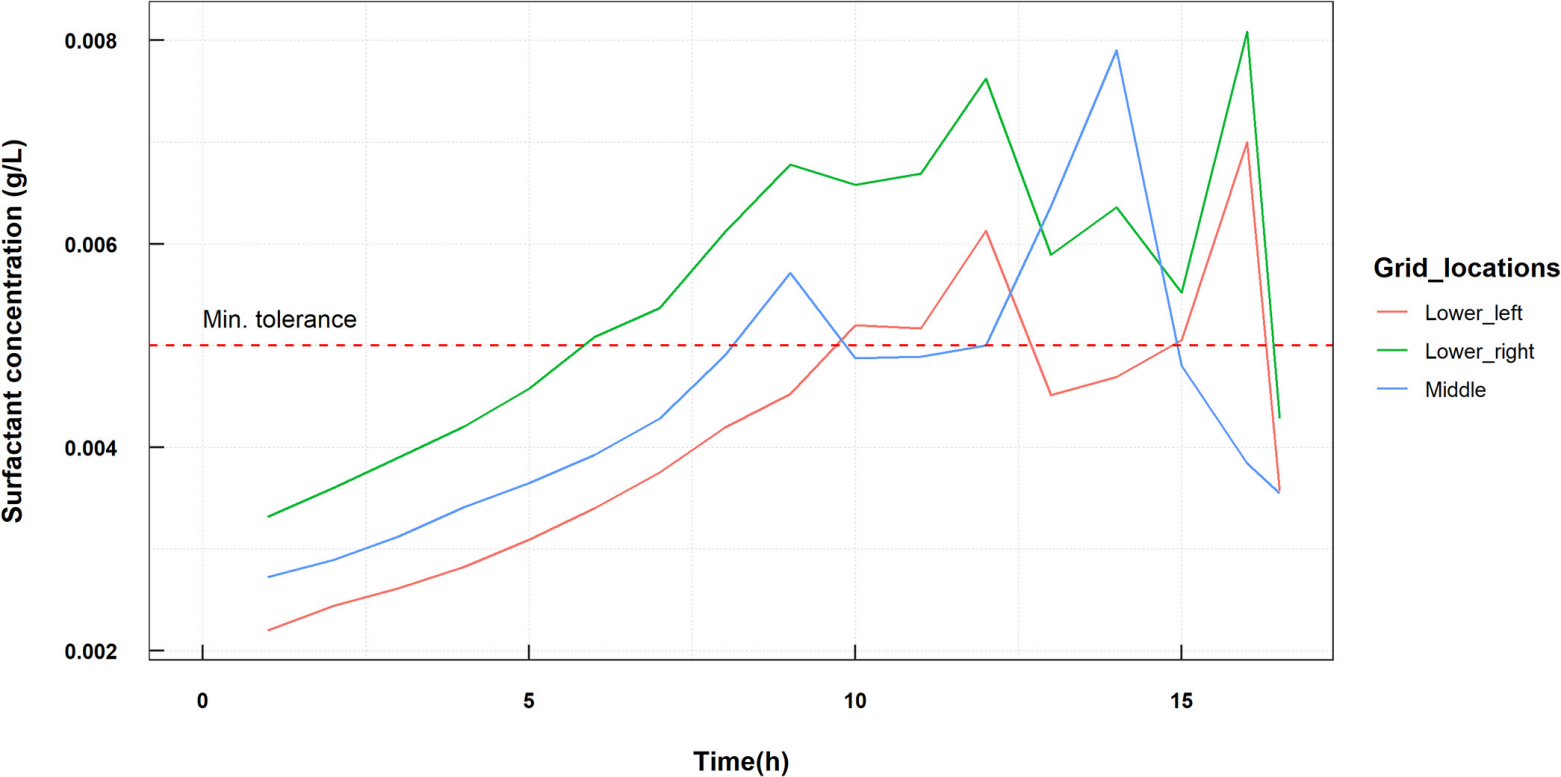
Surfactant concentration in a dual biofilm at different time points in the simulation. Data were sampled at different locations of the biofilm, the lower corners and the center of the surface area covered by the simulation. The red dashed line is the value of 0.005, which is the minimum of the tolerance thresholds for each of the species.

As in the inhibition model, we do not have real data on the tolerance levels of the bacteria to the surfactants. We assume that the *L. paracasei* bacteria require higher concentrations of surfactant before detaching from the biofilm as the biosurfactant is meant to be more antiadhesive to other pathogens (27). The tolerance threshold of surfactant for *L. paracasei* is 0.008 g/liter and for S. oralis is 0.005 g/liter. The thresholds were chosen based on the production of the surfactant by *L. paracasei* and were high enough so as to show a moderate effect (Fig. 6). The surfactant production is given as a separate first-order reaction with *k* = 0.7 (1/h) with a yield of 0.4.

Unlike in the chemotaxis simulation of the work of Sweeney et al. (36), we assume that the planktonic cells do not rejoin the biofilm. To visualize this detachment in the simulation results, detached cells are depicted in a different color than the biofilm cells (Fig. 2E and F). The software includes random movement of planktonic cells in the liquid phase. The modified software that we used is available from https://github.com/alexwweston/iDynoMiCS.

We ran simulations of 16 h of growth for both single- and dual-species biofilms to see the effect of surfactants on the biofilm characteristics. We also ran simulations of the effect of the inhibitory substance and the surfactant together on the dual-species biofilms. In all cases, statistics are provided for multiple simulations with the same input parameters, due to the stochastic nature of the simulations. The number of simulations for the competition and inhibition model was five and for the models with the surfactant was 50. We chose the number of simulations to achieve a coefficient of variation value of 10%. The coefficient of variation was estimated for species biovolume and count.

For each of the simulation models, the biovolume and species count were estimated using modified functions from the R package iDynoR (66). The average biovolumes of the single and dual biofilms were compared using the Games-Howell test in the R package ggstatsplot (67).

### Data availability.

All the relevant code and the iDynoMiCS protocol files for the different models can be obtained from https://github.com/skoshyc/StrepLactoBiofilmModeling.
