# Correction for Archambault et al., “Understanding Lactobacillus paracasei and Streptococcus oralis Biofilm Interactions through Agent-Based Modeling”

**DOI:** 10.1128/msphere.00648-22

**Published:** 2023-03-21

**Authors:** Linda Archambault, Sherli Koshy-Chenthittayil, Angela Thompson, Anna Dongari-Bagtzoglou, Reinhard Laubenbacher, Pedro Mendes

## AUTHOR CORRECTION

Volume 6, no. 6, e00875-21, 2021, https://doi.org/10.1128/mSphere.00875-21. After this paper was published, we discovered that the Lactobacillus paracasei ATCC 334 freezer stocks used in these experiments were contaminated. The figures likely impacted by this contamination are Fig. 1C to F, 3, 4, and S1 to S3. We repeated key experiments with a pure *L. paracasei* ATCC 334 culture in order to ensure the accuracy of the scientific record, as appropriate. We replaced Fig. 1C to F, 3, 4, S1, and S2. The only major difference between the new results and those reported previously is that *L. paracasei* biofilms grow considerably less than in the original published paper, although this does not affect the overall conclusions.

The new data continue to support our main observation that *L. paracasei* reduces the growth of Streptococcus oralis. Therefore, the reasoning behind the modeling of growth inhibition and surfactant mechanisms remains valid. It is important to note that the modeling was not fit against the experiments, so that is unaffected. A revised version of the paper that corrects these errors is being published simultaneously (https://doi.org/10.1128/mSphere.00656-22).

