# Supplementary material for: Understanding Lactobacillus paracasei and Streptococcus oralis Biofilm Interactions through Agent-Based Modeling
Source: mSphere. 2021 Dec 15;6(6):e00875-21. doi: 10.1128/mSphere.00875-21 (PMC8673396; doi:10.1128/mSphere.00875-21)
Supplement: TEXT S1 [file msphere.00875-21-s0001.docx]

**Control simulation**

The control simulation or null model considered contained independent substrates for each bacteria. Both the substrates have the same diffusion coefficient and are in the same initial concentrations. Strep feeds on substrate 1 and lacto substrate 2. The relevant iDynoMiCS protocol file in <https://github.com/skoshyc/StrepLactoBiofilmModeling> is strep_lacto_nullmodel.xml

| Biovolume in the two species biofilm | Competing for space (null model) | Competing for space and nutrients (the competition model in the paper) |
| --- | --- | --- |
| Strep | 18550.34 | 18639.88 |
| Lacto | 8519.99 | 8523.989 |

It was observed that the two bacteria are primarily competing for space because the results of this control simulation were very similar to those of the simulation with a common substrate.

**Sensitivity analysis**

Sensitivity analysis for the four models- competition, inhibition, surfactant, and inhibition+surfactant was performed and the sensitivity coefficients are shown below.

We use here a scaled sensitivity coefficient which is defined as:

S = $\frac{{dV}/V}{{dP}/P}$

where S is the scaled sensitivity coefficient, V is the variable of interest (such as biovolume or species cell count), and P is the parameter manipulated (such as $\mu_{max}$ of a species). Because S is scaled, when it has the value of 1 it means that the variable changes exactly proportionally with the changes in the parameter; a value of S=0.1 means that the variable changes by 10% of the change in the parameter, etc. In order to estimate the value of S of each variable of interest, we use the following operational definition where the derivatives are estimated based on finite differences:

Let x be the parameter of interest

|  | Variable of interest. The average of *N* simulations is estimated for the table values. |
| --- | --- |
| x1= x – 0.05 × x | a |
| x2= x + 0.05 × x | b |

Where *N*=5 for the competition and inhibition models, *N*=50 for the surfactant and inhibition+surfactant models. The estimate of the sensitivity coefficient ($\hat{S}$) is then calculated by the following equation

$\hat{S}=\frac{\left( a-b \right)/a}{\left( x1-x2 \right)/x1}$

**Competition Model**

The relevant part of the biomass growth equation for each bacteria is $\mu_{max}\frac{S_{g}}{K_{S_{g}}+S_{g}}$

Table 1: Sensitivity coefficients for biovolume and cell count for each species in competition model

|  | *S.oralis*  biovolume | *S.oralis*  cell count | *L. paracasei* biovolume | *L. paracasei*  cell count |
| --- | --- | --- | --- | --- |
| $\mu_{max}$ of *S. oralis* | 2.16 | 2.13 | 0.00399 | -0.213 |
| $K_{S_{g}}$ of *S. oralis* | -0.952 | -0.823 | -0.0249 | 0.0807 |
| $\mu_{max}$ of *L. paracasei* | 0.0473 | 0.0995 | 1.32 | 1.31 |
| $K_{S_{g}}$ of *L. paracasei* | -0.0469 | -0.179 | -0.431 | -0.303 |

For the competition model, it was observed that decreasing the $\mu_{max}$ of one bacteria has negligible effect on the other.

**Inhibition model**

The relevant part of the biomass growth equation for *S. oralis* is $\mu_{max}\frac{S_{g}}{K_{S_{g}}+S_{g}}\frac{K_{I}}{K_{I}+I}$

Table 2: Sensitivity coefficients for biovolume and cell count for each species in the inhibition model.

|  | *S.oralis*  biovolume | *S.oralis*  cell count | *L. paracasei* biovolume | *L. paracasei*  cell count |
| --- | --- | --- | --- | --- |
| $\mu_{max}$ of *S. oralis* | 1.06 | 1.52 | 0.00711 | -0.103 |
| $K_{S_{g}}$ of *S. oralis* | -0.343 | -0.254 | -0.0392 | 0.0173 |
| $\mu_{max}$ of *L. paracasei* | -0.204 | -0.201 | 1.29 | 1.23 |
| $K_{S_{g}}$ of *L. paracasei* | 0.137 | -0.186 | -0.420 | -0.588 |
| $K_{I}$ of *S. oralis* | 0.449 | 0.963 | -0.0429 | -0.165 |
| $k$ of toxin production | -0.312 | -0.240 | -0.0765 | -0.184 |

It was observed that the sensitivity coefficient of *S. oralis* with respect to $\mu_{max}$ was half of the coefficient in the competition model. This can be explained by the effect of the toxin on the growth of *S. oralis*.

**Surfactant model**

Table 3: Sensitivity coefficients for biovolume and cell count for each species in the surfactant model.

|  | *S.oralis*  biovolume | *S.oralis cell* count | *L. paracasei* biovolume | *L. paracasei cell*  count | Total *L. paracasei cell* count-(planktonic+  biofilm) | Total *S. oralis* cell count- (planktonic+  biofilm) |
| --- | --- | --- | --- | --- | --- | --- |
| $\mu_{max}$ of *S. oralis* | 7.11 | 7.003 | -0.908 | -0.953 |  |  |
| $K_{S_{g}}$ of *S. oralis* | 4.89 | 5.02 | 1.06 | 0.926 |  | -0.0114 |
| $\mu_{max}$ of *L. paracasei* | -2.66 | -2.60 | -0.450 | -0.383 | 0.301 |  |
| $K_{S_{g}}$ of *L. paracasei* | 0.604 | 0.668 | 0.866 | 0.744 | -0.0459 |  |
| Surfactant tolerance of *S. oralis* | 73.2 | 74.9 | 0.465 | 0.522 |  |  |
| Surfactant threshold of *L. paracasei* | -8.20 | -8.16 | 0.739 | 0.722 |  |  |
| $k$of surfactant production | -2.96 | -2.750 | -1.21 | -1.16 |  |  |

In the surfactant model, the expected behavior of $\mu_{max}$ of *L. paracasei* which is a positive sensitivity coefficient was not observed for the biovolume or count of the *L. paracasei* biofilm cells. The sensitivity coefficient for the planktonic+biofilm cells was positive.

**Inhibition+surfactant model**

Table 4: Sensitivity coefficients for biomass and cell count for each species in the inhibition+surfactant model.

|  | *S.oralis*  biovolume | *S.oralis*  count | *L. paracasei* biovolume | *L. paracasei*  count | Total *L. paracasei* count- planktonic+  biofilm | Total *S. oralis* count- planktonic+  biofilm |
| --- | --- | --- | --- | --- | --- | --- |
| $\mu_{max}$ of *S. oralis* | -1.05 | -0.845 | -0.001 | -0.127 |  | -0.348 |
| $K_{S_{g}}$ of *S. oralis* | -2.36 | -2.43 | 0.112 | 0.189 |  | -0.302 |
| $\mu_{max}$ of *L. paracasei* | -1.14 | -5.55 | -9.29 | -0.199 | 0.188 |  |
| $K_{S_{g}}$ of *L. paracasei* | 7.82 | 6.89 | 0.289 | 0.184 | -0.0696 |  |
| Surfactant tolerance of *S. oralis* | 22.2 | 23.9 | 0.501 | 0.417 |  |  |
| Surfactant threshold of *L. paracasei* | -8.12 | -8.14 | 0.801 | 0.729 |  |  |
| $k$of surfactant and toxin production | -2.55 | -2.96 | -1.36 | -1.26 |  |  |
| $K_{I}$ of *S. oralis* | -2.76 | -3.12 | -0.266 | -0.235 |  | 0.154 |

In the inhibition+surfactant model, the expected behavior of $\mu_{max}$ of *S. oralis* which is a positive sensitivity coefficient was not observed for the planktonic+biofilm cells. We estimate this is the effect of the combined influence of both the toxin and surfactant on *S. oralis* biofilm cells.
