# Supplementary material for: Understanding Lactobacillus paracasei and Streptococcus oralis Biofilm Interactions through Agent-Based Modeling
Source: mSphere. 2021 Dec 15;6(6):e00875-21. doi: 10.1128/mSphere.00875-21 (PMC8673396; doi:10.1128/mSphere.00875-21)
Supplement: TEXT S2 [file msphere.00875-21-s0002.docx]

**ODD Protocol**

To ensure consistency in the structure of the agent-based models (ABMs) presented in the paper, we describe them here using the updated ODD (Overview, Design, Details) protocol proposed by Grimm et al. (1). Since the models were based on the iDynoMiCS software (2) some details are similar to the original paper. In all the models the two bacterial species are *Streptococcus oralis* and *Lactobacillus paracasei*. Both the Surfactant and Inhibition+Surfactant models are run using a modified version of iDynoMiCS 1.1 software (3).

**ODD of the Competition model**

*1. Purpose*

The purpose of this model is to simulate the behavior of *S. oralis* and *L. paracasei* in a biofilm environment as they compete for space and nutrients.

*2. Entities, state variables, and scales*

The entities and their state variables are:

2.1 Agents: The agents are the two bacterial species and their extracellular polymeric substances (EPS) particulates. The agents are assumed to be spherical and their state variables are radius, biomass (active, inert and EPS), grid location, species type, and biomass growth reactions. The EPS is released by an excretion process. The agents also interact mechanically by shoving each other for space (2).

2.2 Environment: The computational domain is divided into three regions: the bulk compartment, which contains dissolved solutes (glucose and oxygen) that diffuse into the biofilm, the biofilm which contains all the agents, and the substratum to which the biofilm adheres. The domain is of size $136\times136\times136$ $\mu m$. The bulk solute concentrations vary with time due to mass transfer within the biofilm (substrate consumption for growth). There is also erosion of the biofilm cells due to the shear forces in the bulk.

*3. Process overview and scheduling*

During a single global time step, the dynamics governing the bulk compartment and the agents are applied. Different timescales are used for the diffusion of solutes, agent behavior, and erosion. The agent behavior is broken down into smaller time steps to account for growth, decay, death and movement. Further details on the process can be found in Algorithm 1 of Lardon et al. (2).

*4. Design concepts*

4. 1 Basic Principles: The model was designed to observe the effect of two bacterial species competing for space and a growth substrates. The biomass growth equations of each species depend on glucose and oxygen concentrations.

4.2 Emergence: The overall structure of the biofilm is governed by the individual agents interacting with each other and the bulk domain. The composition of the bulk depends on the intake by the agents and diffusion into the biofilm.

4.3 Adaptation: Each agent grows according to the solute concentration in their near vicinity. The reactions are applied for each agent independent of the others.

4.4. Objectives: The only fitness objective for each species is their biovolume and cell count.

4.5 Learning: The agent rules are fixed in time and do not change during a simulation.

4.6 Prediction: This model is able to predict the biofilm structure and composition as well as solute concentrations when two microbial species are competing for space and nutrients.

4.7 Sensing: The agents can sense local solute concentrations for growth.

4.8 Interaction: The agents interact with each other mechanically by shoving. They also interact with the solute concentrations through their growth equations.

4.9 Stochasticity: As in the iDynoMiCS paper (2)

4.10 Collectives: The biofilm community is observed as a collective so as to account for erosion and interaction with the bulk. The state variables of each agent are recorded as lists.

4.11 Observation: All the agent state variables (radius, biomass (active, inert and EPS), grid location, species type) at different time steps are recorded as xml files. The solute concentrations of the bulk compartment are also included.

5. *Initialization*

The model is three-dimensional, with a computational grid of size of $136\times136\times136$ $\mu m$ . The parameters defined by us are in Table 1. All the other parameters were kept at default iDynoMiCS values.

Table 1 Parameter values used for cell growth of the two species in the biofilm simulations.

| Parameter | *S. oralis* | *L. paracasei* |
| --- | --- | --- |
| Initial Glucose concentration  𝑆_𝑔_ (g/L) | 2 | 2 |
| Initial Oxygen concentration  𝑆_𝑜_ (g/L) | 0.0064 | 0.0064 |
| 𝜇_𝑚𝑎𝑥_ (1/hr) for growth | 0.32 | 0.153 |
| 𝐾_𝑆𝑔_ (g/L) | 1.756 | 1.2 |
| 𝐾_𝑆𝑜_ (g/L) | 0.192e-3 | 0.2e-3 |
| Yield_glucose | -3 | -0.17 |
| yield_oxygen | -2 | -1 |
| biomass:capsule | 0.8:0.2 | 0.9:0.1 |

All biofilm simulations were started with a seed of 176 cells. In the case of mixed species biofilm simulations, there were 88 cells of each type, such that the initial total number of cells is kept constant. This matches the initial density of 0.01 cells/µm^2^ in the experiments.

6. *Input*

All the iDynoMiCS xml protocol files can be obtained from <https://github.com/skoshyc/StrepLactoBiofilmModeling>. For this model, the relevant protocol files are lacto_single.xml, strep_single.xml, strep_lacto_competition.xml.

7. *Submodels*

All the submodels are as in (2).

**ODD of the Inhibition model**

*1. Purpose*

The purpose of this model is to simulate the behavior of *S. oralis* and *L. paracasei* in a biofilm environment as they compete for space and nutrients, and when *L. paracasei* secretes an inhibitor to the growth of *S. oralis*.

*2. Entities, state variables, and scales*

The entities and their state variables are:

2.1 Agents: The agents are the two bacterial species and their EPS particulates. The agents are assumed to be spherical and their state variables are radius, biomass (active, inert and EPS), grid location, species type, and biomass growth reactions. The biomass growth equation for *S. oralis* also contains the effect of the inhibitory substance secreted by *L. paracasei*. There is also a first order equation for *L. paracasei* for the production of the inhibitory substance. The EPS is released by an excretion process. The agents also interact mechanically by shoving each other for space (2).

2.2 Environment: The environment is as in the Competition model above with the addition of the inhibitory substance produced by *L. paracasei* in the biofilm environment.

*3. Process overview and scheduling:* As in the Competition model.

*4. Design concepts*

4. 1 Basic Principles: The model was designed to observe the effect of two bacterial species competing for space and a sugar source as well as the effect of an inhibitory substance produced by one species on the other. The biomass growth equations of the two species depend on glucose and oxygen concentrations. In the case of *S. oralis*, the growth equation also includes the effect of the inhibitory substance. There is also a first order equation for *L. paracasei* for the production of the inhibitory substance.

4.2 Emergence: As in the Competition model.

4.3 Adaptation: As in the Competition model.

4.4. Objectives: As in the Competition model.

4.5 Learning: As in the Competition model.

4.6 Prediction: This model is able to predict the biofilm structure and composition as well as solute concentrations due to two primary mechanisms. The mechanisms are competition for space and nutrients as well as inhibition of growth of one due to a substance produced by the other.

4.7 Sensing: The agents can implicitly sense local solute concentrations for growth. The agents of the strep species can also sense the concentration of the inhibitory substance produced by the lacto species.

4.8 Interaction: As in the Competition model.

4.9 Stochasticity: As in the Competition model.

4.10 Collectives: As in the Competition model.

4.11 Observation: As in the Competition model.

5. *Initialization*

As in the Competition model. The additional parameters specific to the Inhibition model are in Table 2.

Table 2 Additional Parameters of Inhibition model, all other parameters as specified in Table 1

| Parameter | *S. oralis* | *L. paracasei* |
| --- | --- | --- |
| 𝐾_𝐼_ (g/L) | 0.0025 |  |
| k (1/hr) of production of inhibitory substance by *L.*  *paracasei* |  | 0.7 |
| Yield of inhibitor by *L.*  *paracasei* (g/g) |  | 0.3 |

6. *Input*

All the iDynoMiCS xml protocol files can be obtained from <https://github.com/skoshyc/StrepLactoBiofilmModeling>. For this model, the relevant protocol files are lacto_single.xml, strep_single.xml, strep_lacto_inhibition.xml.

7. *Submodels*

As in the Competition model.

**ODD of the Surfactant model**

*1. Purpose*

The purpose of this model is to simulate the behavior of *S. oralis* and *L. paracasei* in a biofilm environment as they compete for space and nutrients and when *L. paracasei* secretes a surfactant. The surfactant causes the biofilm cells of both species to become planktonic.

*2. Entities, state variables, and scales*

The entities and their state variables are:

2.1 Agents: The agents are the two bacterial species in biofilm and planktonic state. The agents are assumed to be spherical and their state variables are radius, biomass (active and inert), grid location, species type, and biomass growth reactions. There is also a first order equation for *L. paracasei* for the production of the surfactant. The agents also interact mechanically by shoving each other for space (2). The agents also detach from the biofilm and become planktonic based on surfactant concentration (3).

2.2 Environment: The environment is as in the Competition model with the addition of the surfactant produced by *L. paracasei* in the biofilm environment.

*3. Process overview and scheduling*

As in the Competition model with the additional behavior of the biofilm cells becoming planktonic when the surfactant concentration in their vicinity exceeds a threshold value (3).

*4. Design concepts*

4. 1 Basic Principles: The model was designed to observe the effect of two bacterial species competing for space and a carbon substrate source as well as the effect of a surfactant produced by one species that acts on both species. The biomass growth equations of each species depend on carbon substrate and oxygen concentrations. There is also a first order equation for *L. paracasei* for the production of the surfactant.

4.2 Emergence: As in the Competition model.

4.3 Adaptation: As in the Competition model. In addition to growth, the biofilm cells also detach and become planktonic based on the surfactant concentration.

4.4. Objectives: As in the Competition model.

4.5 Learning: As in the Competition model.

4.6 Prediction: This model is able to predict the biofilm structure and composition as well as solute concentrations due to two primary mechanisms, and the amount of planktonic cells. The mechanisms are competition for space and nutrients as well as the effect of surfactant on each species (detachment of cells when surfactant concentration exceeds a threshold). The surfactant is produced by one species.

4.7 Sensing: The agents can implicitly sense local solute concentrations for growth. The agents of both species can also sense the concentration of surfactant.

4.8 Interaction: As in the Competition model.

4.9 Stochasticity: As in the Competition model.

4.10 Collectives: As in the Competition model.

4.11 Observation: As in the Competition model.

5. *Initialization*

As in the Competition model. The additional parameters specific to the Surfactant model are in Table 3.

Table 3 : Additional Parameters of Surfactant model, all other parameters as specified in Table 1.

| Parameter | *S. oralis* | *L. paracasei* |
| --- | --- | --- |
| k (1/hr) of production of inhibitory substance by *L.*  *paracasei* |  | 0.7 |
| Yield of surfactant by *L.*  *paracasei* (g/g) |  | 0.4 |
| Minimum Surfactant concentration required for bacteria to detach (g/L) | 0.005 | 0.008 |

6. *Input*

All the iDynoMiCS xml protocol files can be obtained from <https://github.com/skoshyc/StrepLactoBiofilmModeling>. For this model, the relevant protocol files are lacto_single.xml, strep_single.xml, strep_lacto_surfactant.xml.

7. *Submodels*

As in the Competition model.

**ODD of the Inhibition+Surfactant model**

*1. Purpose*

The purpose of this model is to simulate the behavior of *S. oralis* and *L. paracasei* in a biofilm environment as they compete for space and nutrients and when *L. paracasei* secretes a surfactant and an inhibitory substance. The surfactant causes the biofilm cells of both species to become planktonic. The inhibitory substance slows the growth of *S. oralis*.

*2. Entities, state variables, and scales*

The entities and their state variables are:

2.1 Agents: The agents are the two bacterial species in biofilm and planktonic state. The agents are assumed to be spherical and their state variables are radius, biomass (active and inert), grid location, species type, and biomass growth reactions. There is also a first order equation for *L. paracasei* for the production of the inhibitory substance and another for the production of the surfactant. The agents also interact mechanically by shoving each other for space (2). The agents can also transition to a planktonic phase based on local surfactant concentration (3).

2.2 Environment: The environment is as in the Competition model with the addition of the surfactant and inhibitory substance produced by *L. paracasei* in the biofilm environment.

*3. Process overview and scheduling:* As in the Surfactant model.

*4. Design concepts*

4. 1 Basic Principles: The model was designed to observe the effect of: two bacterial species competing for space and a carbon substrate, an inhibitory substance produced by one species on the other, and a surfactant produced by one species on both species. The biomass growth equations of the two species depend on glucose and oxygen concentrations. There is also a first order equation for *L. paracasei* for the production of the inhibitory substance and the surfactant.

4.2 Emergence: As in the Competition model.

4.3 Adaptation: As in the Competition model.

4.4. Objectives: As in the Competition model.

4.5 Learning: As in the Competition model.

4.6 Prediction: This model is able to predict the biofilm structure and composition as well as solute concentrations due to three primary mechanisms. The mechanisms are competition for space and nutrients, effect of an inhibitory substance on one species, and effect of surfactant on both species. The surfactant and inhibitory substances are both produced by the lacto species. The inhibitory substance effects the growth of the strep species. The surfactant affects both species.

4.7 Sensing: The agents can implicitly sense local solute concentrations for growth. The agents of both species can also sense the concentration of surfactant produced by the lacto species. The agents of the strep species can also sense the concentration of the inhibitory substance produced by the lacto species.

4.8 Interaction: As in the Competition model.

4.9 Stochasticity: As in the Competition model.

4.10 Collectives: As in the Competition model.

4.11 Observation: As in the Competition model.

5. *Initialization*

As in the Competition model. The additional parameters specific to the Inhibition+surfactant model are in Table 4.

Table 4 Additional Parameters of Inhibition+Surfactant model, all other parameters as specified in Table 1.

| k (1/hr) of production of inhibitory substance and surfactant by *L.*  *paracasei* |  | 0.7 |
| --- | --- | --- |
| Yield of inhibitor by *L.*  *paracasei* (g/g) |  | 0.3 |
| Yield of surfactant by *L.*  *paracasei* (g/g) |  | 0.4 |
| Minimum Surfactant concentration required for bacteria to detach (g/L) | 0.005 | 0.008 |

6. *Input*

All the iDynoMiCS xml protocol files can be obtained from <https://github.com/skoshyc/StrepLactoBiofilmModeling>. For this model, the relevant protocol files are lacto_single.xml, strep_single.xml, strep_lacto_inhibitionAndsurfactant.xml.

7. *Submodels*

As in the Competition model.

**REFERENCES**

1. Grimm V, Berger U, DeAngelis DL, Polhill JG, Giske J, Railsback SF. 2010. The ODD protocol: A review and first update. Ecological Modelling 221.

2. Lardon LA, Merkey B v., Martins S, Dötsch A, Picioreanu C, Kreft JU, Smets BF. 2011. iDynoMiCS: Next-generation individual-based modelling of biofilms. Environmental Microbiology 13.

3. Sweeney EG, Nishida A, Weston A, Bañuelos MS, Potter K, Conery J, Guillemin K. 2019. Agent-Based Modeling Demonstrates How Local Chemotactic Behavior Can Shape Biofilm Architecture. mSphere 4.
